# The *Haemonchus contortus* kinome - a resource for fundamental molecular investigations and drug discovery

**DOI:** 10.1186/s13071-015-1231-5

**Published:** 2015-12-08

**Authors:** Andreas J. Stroehlein, Neil D. Young, Pasi K. Korhonen, Abdul Jabbar, Andreas Hofmann, Paul W. Sternberg, Robin B. Gasser

**Affiliations:** Faculty of Veterinary and Agricultural Sciences, The University of Melbourne, Parkville, VIC Australia; Structural Chemistry Program, Eskitis Institute, Griffith University, Brisbane, Australia; HHMI, Division of Biology, California Institute of Technology, Pasadena, CA USA

**Keywords:** *Haemonchus contortus*, Protein kinases, Kinome, *Caenorhabditis elegans*, Bioinformatics

## Abstract

**Background:**

Protein kinases regulate a plethora of essential signalling and other biological pathways in all eukaryotic organisms, but very little is known about them in most parasitic nematodes.

**Methods:**

Here, we defined, for the first time, the entire complement of protein kinases (kinome) encoded in the barber’s pole worm (*Haemonchus contortus*) through an integrated analysis of transcriptomic and genomic datasets using an advanced bioinformatic workflow.

**Results:**

We identified, curated and classified 432 kinases representing ten groups, 103 distinct families and 98 subfamilies. A comparison of the kinomes of *H. contortus* and *Caenorhabditis elegans* (a related, free-living nematode) revealed considerable variation in the numbers of casein kinases, tyrosine kinases and Ca^2+^/calmodulin-dependent protein kinases, which likely relate to differences in biology, habitat and life cycle between these worms. Moreover, a suite of kinase genes was selectively transcribed in particular developmental stages of *H. contortus*, indicating central roles in developmental and reproductive processes. In addition, using a ranking system, drug targets (*n* = 13) and associated small-molecule effectors (*n* = 1517) were inferred.

**Conclusions:**

The *H. contortus* kinome will provide a useful resource for fundamental investigations of kinases and signalling pathways in this nematode, and should assist future anthelmintic discovery efforts; this is particularly important, given current drug resistance problems in parasitic nematodes.

**Electronic supplementary material:**

The online version of this article (doi:10.1186/s13071-015-1231-5) contains supplementary material, which is available to authorized users.

## Background

The decoding of the genome sequence of the free-living nematode, *Caenorhabditis elegans*, in 1998 [1] marked a dawn of the molecular sciences (“-omics”) of multicellular (metazoan) organisms, and the genomes of the fruit fly and human rapidly ensued in 2000 and 2001, respectively [2, 3]. The advent and application of second-generation (short-read) nucleic acid sequencing technology a decade ago [4] then led to a sudden and exponential increase in the amount of genomic and transcriptomic metadata for metazoans, including draft genomes and transcriptomes for numerous parasitic worms (cestodes, trematodes and nematodes) (e.g., [5–16]). However, the bioinformatic “bottleneck” [17] has substantially slowed the processing, annotation and curation of these digital data sets, limiting their conversion into biologically meaningful information and biotechnological outcomes (e.g., drugs and vaccines), such that there is a need to establish and use improved and faster bioinformatics approaches.

Gaining deep insights into molecular pathways of socioeconomically important parasitic nematodes has major implications for developing new interventions against the diseases that they cause in humans, animals or plants (e.g., [7, 12, 18–20]), because it should be possible to define targets in these pathways for the design of new anthelmintics. This aspect is of pivotal importance, because, often, only a limited panel of anthelmintic compound classes are available and used for the treatment of disease/infection, with some having a narrow spectrum of activity, and, importantly, because drug resistance, particularly in gastrointestinal nematodes of animals, has become a major scourge and economic burden to livestock producers [21, 22].

The recent characterisation of the draft genomes and transcriptomes of the barber’s pole worm (*Haemonchus contortus*) [11, 12], one of the most pathogenic nematodes of small ruminants (e.g., sheep and goats) worldwide [23], provides, for the first time, a solid foundation for detailed explorations of molecular pathways amenable to drug target discovery in a nematode that represents many species of a large order (Strongylida) of socioeconomically important pathogens. In addition, the relatively close relatedness of *H. contortus* with *C. elegans* [24, 25], now arguably the best characterised metazoan organism [26], enables direct and detailed comparative analyses of such pathways. Of particular significance in this context are signalling pathways, because of their crucial roles in a plethora of developmental and physiological processes. Many such pathways are regulated by protein kinases, which are enzymes (transferases) that phosphorylate a substrate by transferring a phosphoryl group from an energy-rich molecule, such as adenosine triphosphate (ATP), to a target protein [27]. These kinases are classified into key groups (*n* = 9), families and subfamilies, based on sequence similarity in their catalytic domains and the presence of accessory domains [28–30].

Although there is scant functional information on protein kinases for parasitic nematodes on a genome-wide level, the kinome (i.e. the complement of kinases encoded in the genome) of *C. elegans* is very well characterised and has been functionally investigated [26, 30–32], which provides an ideal starting point for exploring the kinome of *H. contortus* and related nematodes of the order Strongylida. To this end, the aims of the present study were to: (i) predict and curate the full complement of kinases in *H. contortus*, (ii) define transcription levels for kinase genes in all key developmental stages of this parasite; and (iii) prioritise a panel of kinases as drug target candidates, as well as predict chemicals that might bind to these targets, using a practical and effective bioinformatic workflow. Finally, the results of this investigation are discussed in the context of nematode biology and drug discovery.

## Methods

### Defining the *H. contortus* kinome

We used all published transcriptomic and genomic data for *H. contortus* [11, 12] to define the kinome via eight steps (1–8):

1. We used the program getorf (within the EMBOSS package v.6.4.0.0) [33] to identify the open reading frames (ORFs) for all 167,013 transcripts from assembled transcriptomes [12], and retained all non-overlapping ORFs from all six frames with a length of > 100 nucleotides (nt).
2. Using amino acid sequences predicted from these ORFs, we used the program InterProScan v.5.7–48.0 [34] to infer protein domains, families and superfamilies using Pfam v.27.0 [35], PANTHER v.9.0 [36] and SUPERFAMILY v.1.75 [37], respectively. Based on this information, we then identified transcripts encoding complete or partial kinase sequences.
3. We used the assembly program CAP3 (90 % sequence identity over the alignment) [38] to splice associated transcripts. Then, we used the program CD-HIT-EST v.4.6 [39] to cluster transcripts and remove redundancy, employing a sequence identity threshold of ≥ 90 %.
4. We used the program BLAT v.34x12 [40] to map the transcripts to published genomic sequences for *H. contortus* and, employing pslReps (within BLAT), retained the best-aligned matches.
5. We employed the Integrated Genomics Viewer (IGV) [41] to display these mapping results, which enabled us to manually curate transcripts and also verify that they were full-length. This process also allowed us (in > 99 % of cases) to assign individual full-length transcripts to gene loci on genomic scaffolds.
6. We scrutinised the published *H. contortus* gene set [12] and cross-validated the kinase sequences, inferred based on the transcriptome, to identify additional kinases not represented as full-length transcripts in the transcriptomic assembly.
7. We employed the program Kinannote [42] to classify identified protein kinase sequences. If kinases could not be classified using this approach, we used PSI-BLAST v.2.2.26+ [43], employing an E-value cut-off of 10^−5^, to match *H. contortus* kinases to published *C. elegans* kinase sequences [30] and inferred classifications based on the best match. Furthermore, we employed Pfam, PANTHER and SUPERFAMILY annotations using InterProScan v.5.7–48.0 to describe kinases that did not have a *C. elegans* homolog.
8. We used the program EMBOSS Needle v.6.3.1 [33] to determine pairwise global amino acid sequence identities, similarities and the ratio of aligned positions *versus* gaps between *H. contortus* kinases and their closest homologs (based on PSI-BLAST) in *C. elegans* (KinBase) [44], sheep (KEGG) [45], *Dictyocaulus filaria* [46] and *Teladorsagia circumcincta* (WormBase ParaSite; PRJNA72569; WBPS3) [47].

### Transcription analysis

We used publicly available RNA-seq data [12] to assess transcription of kinase genes in all key developmental stages (i.e. egg, L1, L2, L3, L4 male, L4 female, adult male and adult female) of *H. contortus*. First, we used the program Trimmomatic [48] (employing the parameters *phred64*, *ILLUMINACLIP:illuminaClipping. fa:2:40:20,LEADING:3*, *TRAILING:3*, *SLIDINGWINDOW:4:20*, *MINLEN:40*) to filter reads and ensure high quality of the reads. Then, we used Bowtie v.2.1.0 [49] to align the reads to nucleotide sequences encoding kinases, and calculated levels of transcription (transcripts per million, TPM) within the software package RSEM v.1.2.11 [50]. We considered kinase genes to be transcribed if at least five read pairs mapped to their coding regions. Transcripts with similar transcription profiles were clustered based on a Euclidean distance matrix using the Ward clustering method (squaring dissimilarities before cluster updating). The number of clusters (*k*) was selected using the rule of thumb $k\approx \sqrt{\frac{n}{2}}$ [51]. Trend lines were calculated using the Lowess regression method [52]. Clusters were manually selected for further assessment based on cluster size, visibility of trends and amount of variation (i.e. average standard deviation of TPM values) in individual stages within the clusters.

### Drug target prediction and prioritisation

Druggable kinases of *H. contortus* were predicted and prioritised, using a ranking approach, in six steps (i–vi):

(i) We excluded all kinase genes that were not transcribed in at least one of the parasitic stages of *H. contortus* (i.e. L4 and adult).
(ii) For all remaining genes, essentiality was inferred by selecting *H. contortus* kinase sequences homologous (BLASTp; *E*-value ≤ 10^−5^) to *C. elegans* kinases with a lethal phenotype upon gene perturbation (RNAi) listed in WormBase [26], and rewarded with a point.
(iii) Kinases were given one additional point if they were associated with a unique KEGG orthologous gene (KO) term within a KEGG pathway, and another point if they had a unique group/family/subfamily classification.
(iv) An amino acid sequence similarity of > 80 % (> 50 % coverage) to a *C. elegans* homolog was given one point and, to reward low sequence similarity to host (*Ovis aries*) kinases, we gave one point to all *H. contortus* kinase sequences that had similarity values within the lower 75 % quantile (i.e. ≤ 41.45 % sequence similarity) to their closest sheep homolog.
(v) An additional point was given to kinases that had one or more close orthologs (> 80 % sequence similarity; > 50 % coverage) in two parasitic (strongylid) nematodes of importance in sheep (*D. filaria* and *Te. circumcincta*).
(vi) All *H. contortus* kinase sequences were then matched to homologous kinase sequences in the databases Kinase SARfari [53] and DrugBank v.4.3 [54] using PSI-BLAST v.2.2.26+, employing an *E*-value cut-off of 10^−30^. If small-molecule effectors of *H. contortus* kinases were inferred (based on sequence similarity to the reported target in the database), such kinases were given one or two additional points for 1–5 or > 5 associated chemicals, respectively. Chemicals in Kinase SARfari were only considered if they met Lipinski’s rule of five [55] and were flagged as “medicinal chemistry-friendly”. In total, 10 points could be assigned to a target, including a maximum of four points awarded for inferred associations with chemicals (Table 1). Thus, we assigned lower overall scores to kinases that had no or only a small number of associated chemicals, reasoning that they will probably not represent targets for which known chemicals can be repurposed. However, we did not reject such kinases *a priori*, but rather appraised them individually as to their potential as novel drug targets.

**Table 1:** Scoring categories used to rank *Haemonchus contortus* kinases and prioritise them as potential drug targets

Scoring category	Score
Transcribed in parasitic stage (L4 and/or adults)	required
Lethal phenotype in *Caenorhabditis elegans* homolog upon RNAi	1
Associated with a unique function in a pathway (“chokepoint”)	1
Unique classification (group/family/subfamily)	1
> 80 % sequence similarity and > 50 % coverage to *C. elegans* homolog	1
≤ 41.45 % sequence similarity to closest homolog in sheep	1
> 80 % sequence similarity and > 50 % coverage to *D. filaria* and/or *Te. circumcincta*	0.5–1
Associated compound in Kinase SARfari	1
Associated compound in DrugBank	1
> 5 associated compound in Kinase SARfari	1
> 5 associated compound in DrugBank	1

Transcription in at least one parasitic stage was required for a kinase to be considered as a target. Similarity to kinase sequences of one or two related strongylid nematodes (*Dictyocaulus filaria* and/or *Teladorsa gia circumcincta*) of sheep was rewarded with half a point per species. All other categories were given one point

## Results

### The *H. contortus* kinome

In total, 432 *H. contortus* full-length transcripts encoding protein kinases were identified, 428 (99 %) of which were detected in the draft genome. The 432 predicted proteins represented all nine recognised kinase groups and atypical kinases, 103 distinct families and 98 subfamilies. The number of kinases in the kinome of *H. contortus* was similar to that of *C. elegans* (*n* = 434) [44], and for most kinases (*n* = 409, 95 %), we detected a homolog in *C. elegans*, with average overall amino acid identity and similarity values of 35 % and 46 %, respectively. The numbers of kinases for individual groups were similar to those of *C. elegans*, with the exception of the groups CK1, “Other” and TK (Table 2). In the CK1 group, 28 kinases belonged to the “nematode-specific” Worm6 family in both species; except for one Worm10 kinase, members of the other “Worm” families in this group (Worm7 to Worm11) were not present in *H. contortus*. Similarly, only two members (Worm1 and Worm3) of nematode-specific families (Worm1 to Worm5) in the “Other” group were present in *H. contortus*. In contrast, *C. elegans* encodes 11 kinases that belong to these families. Here, the TK group is also greatly expanded in the FER and KIN16 families, with 38 and 15 members, respectively [30]. In *H. contortus*, these numbers are lower, with only 28 members in the FER family and two KIN16 kinase members. In contrast, we found more kinases in some families and subfamilies (DAPK, PKD and NuaK) within the CAMK group of *H. contortus* compared with *C. elegans* (Table 2; Additional file 1: Table S1). This result can be partly explained by a larger number (*n* = 8) of kinase genes in this latter group, predicted to encode multiple isoforms, compared with only one CAMK gene in *C. elegans*, for which two isoforms (R02C2.1 and R02C2.2) exist.

**Table 2:** Characteristics of the *Haemonchus contortus* kinome, and comparison with the kinome of *Caenorhabditis elegans*

Kinase group	Number of *Haemonchus contortus* kinases	Number of *Caenorhabditis elegans* kinases
CMGC	43	48
CAMK	60*	40
AGC	41	29
Other	46*	67
TK	56*	84
TKL	24	15
STE	27	24
CK1	61*	83
RGC	28	27
Atypical	23	17
Unclassified	23	0
Total	432	434

Groups that differ by more than 20 kinases between the two species are marked (*). Figures given here include isoforms (Additional file 1: Table S1)

Interestingly, we identified 23 kinase sequences that appeared to be unique to *H. contortus* and could not be classified. However, during the process of functionally annotating all kinase sequences and linking them to conserved domains, functional categories and molecular pathways (Additional file 1: Table S1), we were also able to gain additional knowledge about these 23 unclassified sequences. Specifically, 16 of them could be assigned to the protein kinase-like superfamily (SSF56112, IPR011009) and were predicted to have phospho-transferase activity (GO:0016772), and 14 were associated with the PANTHER family “uncharacterized nuclear hormone receptor-related” (PTHR23020). Notably, almost all proteins present in the PANTHER database with this annotation were encoded in nematode genomes (only one gene is found in some bacteria and fungi), with the majority reported for *Pristionchus pacificus* (*n* = 44), *C. elegans* (*n* = 31) and *C. briggsae* (*n* = 21), suggesting that these proteins are unique to the phylum Nematoda. Based on other evidence from InterProScan, these same 14 sequences were annotated as “uncharacterised oxidoreductase Dhs-27” (IPR012877), containing a region associated with the CHK region in kinases, and identified a “domain of unknown function, DUF1679” (PF07914). Three other unclassified sequences were annotated as “5’-AMP-activated protein kinase beta-subunit” (PTHR10343).

The functional annotation of all remaining kinases (*n* = 409) showed that most of them matched one of the two profiles representing the catalytic domains of kinases (“protein kinase”, PF00069, *n* = 268; “tyrosine kinase”, PF07714, *n* = 90) and/or were assigned to protein families, such as “protein kinase-like” (SSF56112; *n* = 381), “casein kinase-related” (PTHR11909; *n* = 118), “tyrosine-protein kinase” (PTHR24418; *n* = 68) and/or “adenylate and guanylate cyclases” (PTHR11920; *n* = 56). The most frequently assigned InterPro signatures were “protein kinase-like domain” (IPR011009; *n* = 381), “protein kinase domain” (IPR000719; *n* = 268) and “serine-threonine/tyrosine-protein kinase catalytic domain” (IPR001245; *n* = 90). We also assigned functional annotations (GO terms) to the kinase sequences, including “protein phosphorylation” (GO:0006468; *n* = 387), “transferase activity, transferring phosphorus-containing groups” (GO:0016772; *n* = 381), “protein kinase activity” (GO:0004672; *n* = 358), “ATP-binding” (GO:0005524; *n* = 304), and/or “protein-binding” (GO:0005515; *n* = 77). In addition, a subset of kinases (*n* = 137; 32 %) could be assigned to one or more biological (KEGG) pathways, mainly associated with functions in the categories “organismal systems” (*n* = 80), “cellular processes” (*n* = 79), “signal transduction” (*n* = 75) and “environmental information processing” (*n* = 75). Following this functional annotation, we compared the kinome of *H. contortus* to the draft kinome (514 kinases) of *O. aries* (mammalian host). The number of kinases that had a homolog in *O. aries* (*n* = 403) was only slighty less than that of *C. elegans* (*n* = 409). However, the kinase sequences from *H. contortus* shared substantially less average overall pairwise amino acid sequence identity (24 %) and similarity (35 %) with homologs from sheep than from *C. elegans* (35 % and 46 %, respectively).

### Transcription profiles

We investigated the transcription of kinase genes throughout the life cycle of *H. contortus* in all key developmental stages (egg, L1, L2, L3, L4 and adult) and both sexes (for L4 and adult) (Additional file 1: Table S2). First, we estimated transcription levels of kinase genes for individual groups, which revealed high transcription for a set of CK1 kinase genes in the male L4 and adult stages but low or no transcription in the egg, L1, L2, L3 and female stages (Additional file 2: Figure S1). For all other kinase groups, we observed a slight trend toward increased transcription in the egg and L3 stages (Additional file 2: Figure S1).

Hypothesising that kinases with a similar transcription profile are more likely to function together in the same pathway or to participate in a common signalling network, we assigned kinase transcripts to 15 individual clusters based on similarity in transcription profiles across all developmental stages (Additional file 2: Figure S2). This result indicated that kinase genes in individual clusters were transcriptionally co-regulated in *H. contortus*.

**Fig. 1:**
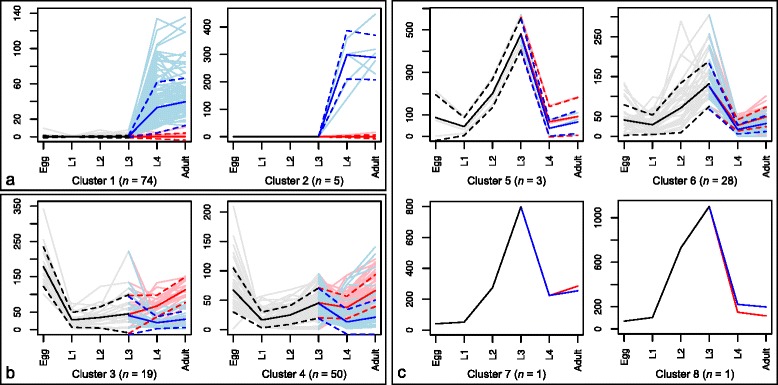
Selected clusters of transcription profiles for kinase genes based on the Ward-clustering method (*k* = 15). Male-enriched clusters (**a**), egg-enriched clusters (**b**) and L3-enriched clusters (**c**) are shown. Transcription levels are represented as transcripts per million (TPM) values on the y-axis (scaled individually according to the highest value within each cluster) and developmental stages (egg, L1-L4 and adult) of *Haemonchus contortus* are shown on the x-axis. Shaded lines represent individual transcription profiles; bold lines represent the Lowess trend line ± standard deviation (dashed lines). For the L4 and adult stages, both sexes are plotted (red = female; blue = male). The complete set of 15 clusters is shown in Additional file 2: Figure S2

Two clusters (Fig. 1a; Additional file 2: Figure S2; clusters 1 and 2) represented transcription profiles with moderate to very high transcription levels (TPM range 10.28–446.40) in *H. contortus* males (L4 and adult) and low or no transcription (TPM range 0.00–7.77) in all other developmental stages studied, with the exception of three genes that were also moderately transcribed in *H. contortus* females (TPM range 10.78–33.36). In total, clusters 1 and 2 contained 79 transcripts, most (*n* = 44) of which represented the CK1 group. Additionally, these clusters contained 23 kinases of the TK group, two members of the “Other” group and five members of each of the CAMK and CMGC groups, respectively. The majority of the sequences representing the CK1 group were further classified into nematode-specific families (TTBKL, Worm6 and Worm10). Of the kinases representing the TK group, 22 belonged to the FER family. Three of five CAMK kinases represented testis-specific serine kinases (TSSK family), and four of five CMGC kinases belonged to the GSK family.

Subsequently, we studied two clusters of a total of 69 genes with high average transcription levels in the egg stage and low to moderate levels in all other developmental stages (Fig. 1b; Additional file 2: Figure S2; clusters 3 and 4). These clusters included homologs of *C. elegans* kinase genes required for cell-cycle progression (e.g., *cdk-1*, *cdk-4* and *chk-1*), embryonic development (e.g., *efk-1*, *hpk-1*, *let-502*, *pat-4*, *pkc-3*, *spk-1* and *zyg-1*), including genes encoding germinal centre kinases (e.g., *gck-1* and *mig-15*) and one gene encoding a kinase linked to chromosome segregation and cytokinesis (*air-2*) [26].

Four other clusters (Fig. 1c; Additional file 2: Figure S2; clusters 5–8) represented 33 sequences that showed high transcription in the L3 stage and considerably lower transcription in all other stages/sexes. Within these four clusters, we found homologs of *C. elegans* genes known to be involved in functions, such as thermosensation (*gcy-23*) [56], suppression of development of vulva and spicules, as well as ovulation (*ark-1*) [57], stress response due to starvation (*mek-1*) [58] and sensory signalling linked to dauer entry/exit (*pdk-1*) [59]. Most kinase genes in the seven other clusters (clusters 9–15) were transcribed relatively uniformly throughout all developmental stages and sexes (Additional file 2: Figure S2).

### Kinases with potential as drug targets

We investigated 405 kinases, whose genes were transcribed in at least one of the parasitic life stages of *H. contortus* (i.e. L4 and adult), as potential anthelmintic targets. A total of 91 kinase sequences matched a *C. elegans* homolog that has a lethal phenotype upon gene perturbation (RNAi; Additional file 1: Table S1). Furthermore, 64 kinases encoded in *H. contortus* represented metabolic “chokepoints”, i.e. they could be assigned a unique function in a pathway. In addition, 103 kinases were assigned to unique families or subfamilies. Of all 405 sequences, 25 had close homologs in *C. elegans*, 86 had close homologs in one or both of the strongylid nematodes *D. filaria* and *Te. circumcincta*, and 300 lacked a close homolog in sheep. Subsequently, we interrogated drug databases to infer all chemicals associated with the 405 putative kinase targets. We matched 239 kinases with 509 chemicals (1–114 per kinase) in DrugBank, and 191 kinases with 22,861 chemicals (2–10,186 per kinase) in Kinase SARfari.

**Fig. 2:**
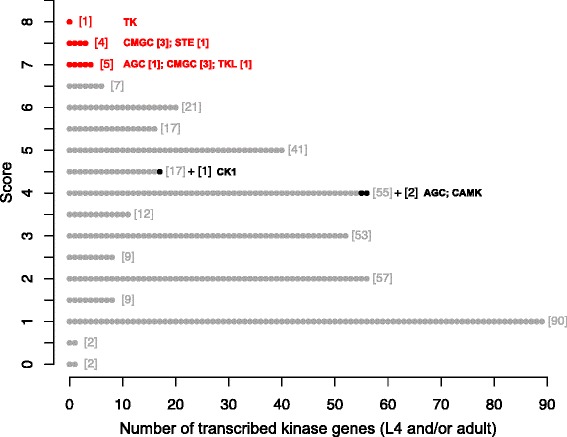
Prediction of druggable kinases of *Haemonchus contortus* based on ranking score. Kinases prioritised as either “repurposing” targets or novel targets are highlighted in red and black, respectively. The group classification is indicated for each kinase; the total numbers of kinases with a particular score are indicated in square brackets

We then ranked all of these 405 kinases according to their potential as drug targets (Fig. 2; cf. Table 1). Ten kinases had a ranking score of ≥ 7, including six CMGC kinases and one AGC, STE, TK and TKL kinase, respectively. A large number of small-molecule compounds were associated with these 10 highest-ranked targets in Kinase SARfari (*n* = 3105) and DrugBank (*n* = 122). We excluded compounds that were predicted to non-selectively bind to a range of kinases from these two sets, and retained 1391 (Kinase SARfari) and 122 (DrugBank) chemicals that were exclusively associated with one or more of the top-ten targets (Table 3; Additional file 1: Tables S3 and S4). Then, we appraised the scores of all remaining kinases (*n* = 395) to identify novel target candidates that associated with no or only a small number of chemicals. Thus, we identified three additional kinases (AGC, CAMK and CK1; black dots in Fig. 2; Table 3) that had a score of 4 or 4.5, but had only been given one additional point for an associated compound (eight chemicals in total). Taken together, we prioritised 13 kinases of *H. contortus* (from seven distinct groups, 10 families and 11 subfamilies) as druggable targets (Table 3).

**Table 3:** Ten prioritised (highest-scoring) kinase targets in *Haemonchus contortus*, and three predicted novel targets

*H. contortus* kinase identifier	Closest *C. elegans* homolog	Description [90]	Classification	Score	Number of associated compounds in DrugBank/Kinase SARfari
*Hc*-PK-358.1	CSK-1	C-terminal Src kinase	TK/CSK	8	12/735
*Hc*-PK-062.1	CDK-12	Cyclin-dependent kinase	CMGC/CDK/CRK7	7.5	17/0
*Hc*-PK-063.1	CDK-5	Cyclin-dependent kinase	CMGC/CDK/CDK5	7.5	7/655
*Hc*-PK-236.1	CDK-9	Cyclin-dependent kinase	CMGC/CDK/CDK9	7.5	1/655
*Hc*-PK-197.1	SEK-1	SAPK/ERK kinase	STE/STE7/MEK3	7.5	10/0
*Hc*-PK-263.1	WTS-1	WarTS (*Drosophila*) protein kinase homolog	AGC/NDR/LATS	7	4/0
*Hc*-PK-002.1	CDK-7	Cyclin-dependent kinase	CMGC/CDK/CDK7	7	2/0
*Hc*-PK-199.1	GSK-3	Glycogen synthase kinase	CMGC/GSK	7	19/1
*Hc*-PK-210.1	LIT-1	“Loss of intestine”; serine - threonine protein kinase	CMGC/MAPK/NMO	7	14/0
*Hc*-PK-261.1	DLK-1	Dual-leucine zipper kinase	TKL/MLK/LZK	7	46/0
*Hc*-PK-165.1*	CSNK-1	Casein kinase	CK1/CK1/CK1-G	4.5	3/0
*Hc*-PK-092.1*	RSKS-1	Ribosomal protein S6 kinase	AGC/RSK/RSKP70	4	4/0
*Hc*-PK-262.1*	CHK-2	Checkpoint kinase	CAMK/RAD53	4	1/0

The table shows the closest homolog in *Caenorhabditis elegans*, kinase classification, score and number of associated small-molecule compounds in the DrugBank and/or Kinase SARfari databases for each target. Predicted novel targets are marked (*). Individual compound codes can be accessed in Additional file 1: Tables S3 and S4

## Discussion

### The *H. contortus* kinome

Signalling cascades that are regulated by protein phosphorylation events play key roles in all eukaryotic organisms, and investigations of these events in many metazoans, including the free-living nematode *C. elegans*, has helped us gain a sound understanding of how processes, such as growth, development and tissue differentiation, are regulated at the cellular and subcellular levels [29, 30, 60]. In contrast, there is relatively little information on these processes in parasitic nematodes. However, the use of advanced sequencing and computational methods makes it possible to gain deep insights into the genomes and transcriptomes of these worms and, hence, explore their kinomes using bioinformatics tools.

The recent assembly and annotation of draft genomes and transcriptomes of *H. contortus* [11, 12] have provided unique opportunities to explore molecular signalling and other biological pathways in this parasite. Originally, we had predicted 845 sequences from these drafts that shared sequence homology (BLASTp; *E*-value cut-off of ≤ 10^−5^) to kinases [12]. This estimate was higher than for published draft kinomes (*n* = 233–364) of other parasitic nematodes, including *Ascaris suum*, *Brugia malayi*, *Loa loa*, *Meloidogyne hapla*, *Trichinella spiralis* and *Wuchereria bancrofti* [42, 61], as well as the free-living nematode *C. elegans* (*n* = 434) [31], suggesting that we had over-estimated the number of kinases, which is to be expected when sequence homology alone is used for annotation [62]. Therefore, in the present study, our goal was to characterise and curate the complement of kinases of *H. contortus* in detail using a refined bioinformatic workflow that incorporates and extends that described recently [63]. By using this approach and by integrating high-quality transcriptomic data, we overcame some of the challenges associated with the assembly (i.e. fragmentation) and the annotation of a complex, eukaryotic genome (cf. [12]), and thus considerably improved the gene prediction and annotation of protein kinases encoded in *H. contortus*.

The finding that 409 of the 432 kinase sequences identified and classified in the present study have homologs in *C. elegans* is consistent with the relatively close phylogenetic relationship of these two nematodes [25], and contrasts results for the draft kinomes of parasitic nematodes representing different orders or clades that are reported to have substantially reduced kinase complements (*n* = 233–364), presumably having lost (or not gained) particular kinase families during nematode evolution [61]. The reduced number of kinases in parasitic nematodes compared with *C. elegans* might be explained by differences in the environmental conditions that these parasites are exposed to, as well as differences in their lifestyle, but it has also been proposed that the small numbers of kinases of some species (e.g., *M. hapla* and *Tr. spiralis*) might relate to fragmented and/or incomplete genomic assemblies [61]. Future genome sequencing and annotation efforts should allow these proposals to be tested.

The distinctiveness between *H. contortus* and *C. elegans* in the numbers of kinases within four groups (CK1, TK, “Other” and CAMK) are likely associated with differences in biology, habitat and/or life cycle between these two nematodes. For instance, *H. contortus* has a comparatively short free-living phase on pasture and (usually) a longer phase as a haematophagous parasite inside the ruminant host, whereas *C. elegans* completes its entire life cycle and lives in a soil environment. This difference might explain the larger CK1 group in *C. elegans* that has been proposed to associate with an increased need for enhanced DNA repair mechanisms in response to excessive exposure to mutagenic stress in this environment [31].

The reason for a reduced number of kinases in the two families FER and KIN16 (TK group) in *H. contortus* is elusive, but again, likely relates to variation in worm biology. Little is known about the apparent “expansion” (i.e. an increased number) of FER kinases in *C. elegans*, but it might associate with an adaptation of the Wnt signalling pathway (cf. [30]) and/or cell adhesion mechanisms within the nematode, based on the known roles for two mammalian homologs (FER and FES) [31]. Kinases of the KIN16 family are involved in hypodermal development in *C. elegans* [64], and at least one representative of this family (TKR-1) could be linked to an increased resistance against ultraviolet radiation and thermal stress [31, 65]. Similar to expansions in these two tyrosine kinase families, kinases of the “Worm” families in the “Other” group appear to be largely *Caenorhabditis*-specific and not present in other nematodes, including the Strongylida [30, 61]. Clearly, future investigations are warranted to explain the reduced numbers of kinases of these various groups/families in *H. contortus*. Generally, the present findings suggest that expansions in some kinase groups and families in *C. elegans* are recent events, which is further supported by phylogenetic analyses of some of these families (e.g., KIN16), members of which share limited sequence homology between *C. elegans* and its close relative, *C. briggsae* [30].

Several smaller expansions in families and subfamilies (DAPK, PKD and NuaK) within the CAMK group of *H. contortus* might reflect an adaptation in response to environmental stressors (e.g., bacterial pathogens) and/or starvation. This interpretation is supported, to some extent, by the roles that such kinases assume in defence mechanisms and/or in response to starvation, such as apoptosis and autophagy [66–68]. In addition, kinases of the NuaK family are involved in cell adhesion [69], and an expansion of this family might compensate for the reduction of the somewhat smaller FER family in *H. contortus*, which, in *C. elegans*, contains kinases involved in adhesion [31].

Most (*n* = 16) of the 23 kinases for which we did not detect a homolog in the *C. elegans* kinome had homologous sequences in the SwissProt database (data not shown). Six of these 16 kinases were homologs of *C. elegans* YLK1. Thirty members of this kinase-like protein family were originally reported in the first global study of the *C. elegans* kinome, and it was suggested that they likely have kinase activity [31]. However, in the most recent release of the *C. elegans* kinome [44], these kinases are no longer listed, which explains why their *H. contortus* orthologs were not initially identified and classified here. These YLK1-related kinases share sequence homology with bacterial aminoglycoside kinases, enzymes that confer antibiotic resistance [70]. Thus, orthologs of these kinases might play a similar role in defence against microbial agents in *H. contortus* and *C. elegans*. The functions of the seven other *H. contortus* kinases, for which we did not detect a homolog, are currently unknown, but their domain architecture and functional classification indicate that they are indeed protein kinases. These findings warrant future investigations.

Taken together, the finding that the kinase complements of *H. contortus* and *C. elegans* are relatively conserved, provides unique opportunities to explore the functions of these enzymes in *H. contortus* and to gain an improved understanding of the underlying molecular processes/mechanisms that regulate development, reproduction and physiology in this parasite. This focus is of particular importance, given that direct functional investigations of *H. contortus* using RNAi-mediated gene knockdown are not very permissive [71, 72]. By contrast, RNAi is well established in *C. elegans*, and kinase genes can be readily knocked down, allowing spatial and temporal expression to be assessed using transgenic animals containing reporter genes [32].

### Transcriptional regulation of kinase genes in *H. contortus*

During its life cycle, *H. contortus* undergoes substantial transcriptional regulation of genes, which is tightly restricted to particular developmental stages [11, 12, 73–76]. Stage-specific transcription levels suggest critical roles in signalling cascades for a suite of kinase genes/gene products at particular phases of development. For example, many of the 69 highly transcribed kinase genes in the egg stage of *H. contortus* are probably linked to cell-cycle progression, growth, embryogenesis and tissue differentiation, taking place during early development [77–79]. The upward trend of the female transcription values for the two egg-enriched clusters (Fig. 1b ) is likely attributed to eggs being within the uterus of gravid females, a proposal that is supported by the finding that this trend is not seen in the female L4 stage (Fig. 1b). After the L1 hatches from the egg (~1 day), it moults twice within ~1 week and develops, via L2, into the infective larval stage (L3). During this time, L1 and L2 stages actively feed on microbes and continue to grow [80]. Most kinase genes transcribed in the L1 and L2 stages were also relatively uniformly transcribed in all other developmental stages, suggesting that they assume more generalized functions throughout the entire life cycle.

The final free-living and infective larva (L3) is ingested by the host animal, which marks the transition to the parasitic phase of the *H. contortus* life cycle*.* L3s are ensheathed and unable to actively feed, and, therefore, need to live on accumulated reserves at reduced metabolic rates [12, 81]. Their development is arrested, but they remain motile, and various kinases appear to assume crucial functions in this developmental stage. For example, the *Hc-pk-119* gene encoding a MEK7 (family) kinase is likely involved in a stress response to starvation, as has been shown for its *C. elegans* homolog *mek-1* [58]. In addition, several kinase genes that are highly transcribed in the L3 stage seem to function as inhibitors of processes that become important at a later stage of development. For instance, *Hc-pk-088* is predicted to encode a kinase that likely inhibits LET-23*,* an EGF receptor responsible for vulva, ectoderm and spicule development, based on evidence for its *C. elegans* homolog ARK-1 [57]. Another example is PDK-1 (*Hc*-PK-240), which is involved in signalling pathways controlling entry into and exit from dauer [59].

The overall high transcription of many kinase genes in the L3 stage (Fig. 1; Additional file 2: Figure S1) also suggests that transcripts might be stored within this stage to allow for a rapid development and adaptation to a “hostile” environment (pH = 1–2) within the host stomach (abomasum) following exsheathment. The latter process is mainly triggered by increased CO_2_ concentrations and also by the host’s body temperature (38.3–39.9 °C), and it is likely that kinases play a central role in this transition to parasitism. The high transcription of *Hc-pk-282* in the L3 stage, but the lack thereof in L4 and adult stages, for example, suggests one or more specific roles in the L3 exsheathment process in *H. contortus*, a statement that is supported by evidence that a *Hc*-PK-282 homolog in *C. elegans* (GCY-23) is involved in thermosensation [56].

Following exsheathment, L3s undergo a short histotropic phase in the stomach mucosa [82] and then develop to haematophagous stages (L4s and adults) [80]. This development is reflected in a range of morphological changes in both male and female worms, including the formation of a buccal capsule in both sexes, bursal rays and spicules in the male, and development of vulva and other reproductive organs in the female. Particularly in the male, morphogenesis and spermatogenesis appear to be controlled by a range of specific protein kinases (Fig. 1). The majority of kinases represented in the two male-enriched clusters (Fig. 1a) were members of the CK1 group and FER family, including homologs encoded by the two *spe*/*fer* (spermatogenesis-/fertilization-defective) mutants *spe-6* and *spe-8* [30, 83], suggesting a role in spermatogenesis and, more generally, fertility. In addition, protein kinases have also been reported to play crucial roles in the post-translational regulation of nematode sperm development, including pseudopod extension and movement [77]. In addition, the testis-specific serine kinases (TSSKs) and GSKs in the male-specific clusters might have roles in the formation of bursal rays and spicules in *H. contortus*, given that the *C. elegans* homologs are represented in Wnt signalling-related pathways associated with similar anatomical changes [84]. Future studies should elucidate the actual functional roles of kinases and signalling pathways in development and reproduction of *H. contortus*, and assess to what extent these mechanisms differ between parasitic and free-living nematodes; this might be achievable in *H. contortus* by RNAi using a virus-based transduction system (cf. [85]).

### Protein kinases of *H. contortus* as potential drug targets

In addition to the benefits of using *C. elegans* as a tool for comparative functional studies of molecular processes in parasitic nematodes (e.g., [86–89]), there is merit in using information on essential genes/gene products in *C. elegans* (in WormBase) [90] to predict and prioritise new drug targets in *H. contortus* [91]. Currently, the small number of classes of anthelmintics available to treat infections with *H. contortus* and other strongylid nematodes (e.g., [92, 93]) and widespread drug-resistance in these parasites [21, 22] necessitate the search for new nematocidal drugs with modes of action that are distinct from those of currently available classes.

In this context, protein kinases might be suitable targets. In a recent study [12], 27 protein kinases have been proposed as drug target candidates in *H. contortus*, based on lethal RNAi phenotypes of their homologs in *C. elegans* and because they were predicted to assume indispensable functions in molecular pathways (“chokepoints”) in *H. contortus*. In the present study, we applied additional criteria for target prediction/prioritisation (see Table 1) and used a ranking system to define a set of 13 druggable targets and a total of 1517 associated small-molecule compounds. A similar drug target/drug prioritisation strategy has been implemented previously in the TDR database [94]. This “union strategy” has the advantage that it allows the user to adjust the weights for each criterion, and also provides a genome-wide perspective on how useful the chosen criteria and weights may be [19]. However, while the latter database contains information for various “neglected” pathogens (*Mycobacterium*; *Plasmodium*, *Toxoplasma*, *Leishmania*, *Trypanosoma*; *Brugia* and *Schistosoma*) and *C. elegans* (as a reference), it does not presently permit the prioritisation of drug targets and/or chemicals for other pathogens, such as *H. contortus*.

Eleven chemicals listed in DrugBank and associated with the 13 highest-ranking targets identified here were previously identified in a target/compound prioritisation approach and then screened against *C. elegans* and *H. contortus*, of which three of these chemicals (DB02152, DB02010 and DB04707) had an inhibitory effect on both nematodes [95]. The authors of this study selected chemicals based on a set of protein kinases that were conserved among biologically and genetically diverse nematode species (*B. malayi*, *C. elegans*, *M. incognita* and *Tr. spiralis*), with the goal of finding chemicals that have a broad spectrum of activity against many parasitic nematodes. In the present study, we took a similar, but more conservative approach, "rewarding" potential targets with an additional point if they had a close homolog (i.e. > 80 % sequence similarity) in *D. filaria* and *Te. circumcincta*, two related parasites of importance also in small ruminants. This was the case for seven of the 13 top-scoring targets in at least one of these two species, suggesting that these seven kinases are relatively conserved in sequence among all three strongylids and *C. elegans*, and might be inhibited by a single ligand. However, defining inhibitors with broad-spectrum anthelmintic activity against members representing distinct phyla (e.g., Nematoda and Platyhelminthes) might be challenging to achieve, given evidence of major differences in efficacy of some compounds (e.g., DB03693, DB02984 and DB03044) among select members (*C. elegans*, *H. contortus* and *B. malayi*) within the phylum Nematoda [95], thus requiring careful cross-phylum investigations of parasite kinomes.

Interestingly, homologs of four of the kinases (*Hc*-PK-062.1, *Hc*-PK-199.1, *Hc*-PK-210.1 and *Hc*-PK-165.1) that were conserved in sequence among multiple of the four nematode species investigated here have been identified previously in an RNAi screen of *C. elegans* [32] as being essential for protein homeostasis, mitochondrial network structure and/or sarcomere structure in muscle. Therefore, assessing chemicals for their ability to specifically inhibit one or more of these four kinases and disrupt normal muscle formation and/or morphology might represent a promising path; while many of the approved veterinary drugs bind to neuromuscular targets, thereby disrupting normal muscle functionality (e.g., piperazine, pyrantel, morantel, levamisole, ivermectin, emodepside) [93], inhibiting processes controlling muscle formation and/or structure in the parasite might also prove fruitful.

In summary, the prioritised set of potential kinase inhibitors identified here provides a starting point for future, targeted screening on parasitic stages of *H. contortus*. Thus, a subset of the 1517 chemicals could now be selected, according to cost, availability, chemical properties, safety and/or prior use as drugs, and tested for anthelmintic effects in an established, automated, whole-worm motility screening assay [96], followed by a hit-to-lead phase, in which structural analogs of “hit” compounds could be synthesised and screened to establish structure-activity relationships (SARs), and then tested in established assays to predict intestinal absorption, distribution, metabolism, excretion and toxicity (ADMET) [97]. In addition, *C. elegans* could be used as a complementary tool for the validation of targets and their functions, for investigating modes of action of prioritised chemicals and for the prediction of how long it takes for nematodes to develop resistance against such chemicals. Finally, compounds with desired parameters that are metabolically stable and are not cytotoxic to mammalian cells could then progress to initial *in vivo* testing in sheep.

## Conclusions

The *H. contortus* kinome should provide a useful resource for fundamental investigations of signalling pathways in this nematode, and will likely facilitate future drug discovery/repurposing efforts for a variety of parasitic worms. With this in mind, further studies should focus on curating the kinase complements of a range of socioeconomically important parasitic worms using the present bioinformatic workflow system, with a view toward predicting pan-phylum anthelmintic targets.

## Additional files

Additional file 1: Table S1. The *Haemonchus contortus* kinome, orthologs in *Caenorhabditis elegans* and *Ovis aries*, amino acid sequence identities and similarities, functional annotations, and nucleotide and amino acid sequences. **Table S2.** Transcription profiles of 432 *Haemonchus contortus* protein kinase transcripts. **Table S3.** Chemicals in DrugBank associated with the 13 predicted *Haemonchus contortus* kinase drug targets. **Table S4.** Chemicals in Kinase SARfari associated with the 13 predicted *Haemonchus contortus* kinase drug targets. (XLSX 1004 kb) Additional file 2: Figure S1. Transcription profiles for kinase genes in all key developmental stages (egg, L1, L2, L3, L4 and adult) and both sexes (L4 and adult) of *Haemonchus contortus* for eleven individual kinase groups. **Figure S2.** All clusters of transcription profiles for *Haemonchus contortus* kinase genes based on the Ward-clustering method (*k* = 15). (DOCX 222 kb)
