# Supplementary material for: The Haemonchus contortus kinome - a resource for fundamental molecular investigations and drug discovery
Source: Parasit Vectors. 2015 Dec 8;8:623. doi: 10.1186/s13071-015-1231-5 (PMC4672506; doi:10.1186/s13071-015-1231-5)
Supplement: Additional file 2: Figure S1. — Transcription profiles for kinase genes in all key developmental stages (egg, L1, L2, L3, L4 and adult) and both sexes (L4 and adult) of Haemonchus contortus for eleven individual kinase groups. Figure S2. All clusters of transcription profiles for Haemonchus contortus kinase genes based on the Ward-clustering method (k = 15). (DOCX 222 kb) [file 13071_2015_1231_MOESM2_ESM.docx]

**Additional files: Figures S1 and S2**

**Figure S1** **Transcription profiles for kinase genes in all key developmental stages (egg, L1, L2, L3, L4 and adult) and both sexes (L4 and adult) of *Haemonchus contortus* (x-axis) for eleven individual kinase groups (individual panels; abbreviations described below).** Transcription levels are represented as log(transcripts per million + 1) values (y-axis). Shaded lines represent individual transcription profiles; bold lines represent the Lowess trend line ± standard deviation (dashed lines). For the L4 and adult stages both sexes are plotted (red = female; blue = male). CK1 = Casein kinase 1; CMGC = Cyclin-dependent kinases, mitogen-activated protein kinases, glycogen synthase kinases and CDK-like kinases; CAMK = Ca^2+^/calmodulin-dependent kinases; AGC = Nucleoside-regulated kinases; TK = Tyrosine kinases; TKL = Tyrosine kinase-like kinases; STE = MAPK cascade kinases; RGC = Receptor guanylate cyclases; UNCL = Unclassified kinases.

**Figure S2 All clusters of transcription profiles for *Haemonchus contortus* kinase genes based on the Ward-clustering method (*k* = 15).** Transcription levels are represented as transcripts per million (TPM) values (y-axis; scaled according to the highest value within each cluster), and developmental stages (egg, L1-L4, adult) of *H. contortus* (x-axis). Shaded lines represent individual transcription profiles; bold lines represent the Lowess trend line ± standard deviation (dashed lines). For the L4 and adult stages, both sexes are plotted (female = red; male = blue).
